# Decision Support Tool in the Selection of Powder for 3D Printing

**DOI:** 10.3390/ma17081873

**Published:** 2024-04-18

**Authors:** Ewelina Szczupak, Marcin Małysza, Dorota Wilk-Kołodziejczyk, Krzysztof Jaśkowiec, Adam Bitka, Mirosław Głowacki, Łukasz Marcjan

**Affiliations:** 1 Faculty of Metals Engineering and Industrial Computer Science, AGH University of Krakow, al. Mickiewicza 30, 30-059 Kraków, Poland; esz98@wp.pl (E.S.); krzysztof.jaskowiec@kit.lukasiewicz.gov.pl (K.J.); glowacki@metal.agh.edu.pl (M.G.);; 2 Łukasiewicz Research Network—Krakow Institute of Technology, Zakopiańska 73, 30-418 Kraków, Poland; adam.bitka@kit.lukasiewicz.gov.pl; 3 Faculty of Foundry, AGH University of Krakow, al. Mickiewicza 30, 30-059 Kraków, Poland; 4 Faculty of Natural Sciences, Jan Kochanowski University of Kielce, ul. Żeromskiego, 25-369 Kielce, Poland

**Keywords:** 3D printing, XGBoost, machine learning algorithms, random forest, decision tree, K-nearest neighbors, fuzzy K-nearest neighbors, gradient boosting

## Abstract

The work presents a tool enabling the selection of powder for 3D printing. The project focused on three types of powders, such as steel, nickel- and cobalt-based and aluminum-based. An important aspect during the research was the possibility of obtaining the mechanical parameters. During the work, the possibility of using the selected algorithm based on artificial intelligence like Random Forest, Decision Tree, K-Nearest Neighbors, Fuzzy K-Nearest Neighbors, Gradient Boosting, XGBoost, AdaBoost was also checked. During the work, tests were carried out to check which algorithm would be best for use in the decision support system being developed. Cross-validation was used, as well as hyperparameter tuning using different evaluation sets. In both cases, the best model turned out to be Random Forest, whose F1 metric score is 98.66% for cross-validation and 99.10% after tuning on the test set. This model can be considered the most promising in solving this problem. The first result is a more accurate estimate of how the model will behave for new data, while the second model talks about possible improvement after optimization or possible overtraining to the parameters.

## 1. Introduction

Elements manufactured using 3D printing technology are tested in the context of the final product parameters such as tensile strength, hardness and elongation [1,2,3]. In this process, it is important to select the appropriate material, in particular the chemical composition, grain size and type of heat treatment [4,5]. Research is also being conducted to use artificial intelligence methods to optimize parameters and predict the mechanical properties of these 3D printed components [6,7,8,9,10]. Examples of intelligent decision support systems are available in the literature [11,12,13,14,15] for supporting the planning of technological processes of machining and 3D printing and others technology. Artificial intelligence methods (e.g., neural networks) are also used in the context of material selection [16]. With respect to 3D printing technology, SVM support vector machines have been used [17,18,19,20]. It was decided that research would be undertaken as part of the work by considering the results of using algorithms based on artificial intelligence, which would show that the results obtained when using them are helpful in creating decision-making systems supporting the selection of the appropriate material and, as a result, process parameters. The work focuses on developing a practical tool using the results of tested algorithms to support the decision-making process in selecting the best powder for 3D printing, taking into account the specific technical requirements of the product.

## 2. Materials and Methods

The input data in this work is information about composition powders intended for 3D printing using the Selective Laser Melting (SLM) technology (a method involving selective sintering of metal powders, deposited layer by layer, with a laser beam) [2,3,4,5,6,7,8,9,10,11,12,13,14,15,16,17,18,19,20,21,22,23]. The input data includes mechanical properties, chemical composition and material prices. Based on the literature and after consultations with an expert, it was decided that the most important mechanical properties of the products are tensile strength, elongation and Vickers hardness, and these are the focus of this article. Consultations with experts were carried out regarding the need to create a system supporting decision-making in this area, parameters that influence the process of their mutual interaction, and the availability of information in this area. Project work was carried out at SBŁKIT, using the experience of people working on the project “The use of additive manufacturing technology (DLD—Direct Laser Deposition) and SLM technology (Selective Laser Melting) for the development of parts for permanent die molds with increased exploitation parameters used in high-pressure die casting and gravity die casting” that made this type of action possible. When obtaining data on powders, the focus was on three groups of powders: steel-based, nickel- and cobalt-based, and aluminum-based powders. Table 1 presents an example of the collected data, which constituted the input database in the work. The input database contained mechanical property values for 13 different powders that were subjected to various heat treatments, a total of 47 records. It is worth noting that the data on mechanical properties were obtained from the results of experiments, and the result was the average value and standard deviation; therefore, in Table 1, the mechanical properties are presented in the form of ranges (ranges). To increase the amount of data, it was decided to adopt an approach in this work that involves manually generating data based on existing ranges and then using them to train models. For this reason, it should be remembered that the results obtained may differ slightly from the results that could be achieved using only real data, but for the needs of the project and due to the lack of sufficient real data, it was consciously decided to use such an approach. When generating the data, efforts were made to replicate real data as closely as possible to obtain the best possible powder recommendations. The presented methodology was consulted with people with appropriate knowledge and experience in this topic: employees of the Łukasiewicz Research Network, Krakow Institute of Technology.

Additionally, detailed information on the chemical composition of the tested materials was also collected. Thanks to such information, it was possible to program additional functionality of the application, consisting of eliminating powders containing undesirable elements. For example, if the user knows that a material containing copper may be more susceptible to corrosion in an acidic environment, he can select the application to return an appropriate powder that does not contain copper.

### 2.1. Correlation Analysis of the Dataset

**Figure 1:**
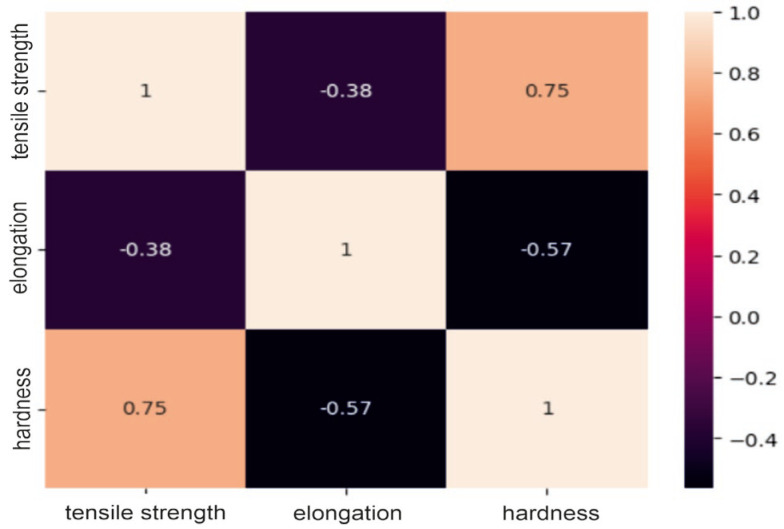
Correlation matrix for a group of aluminum-based powders.

**Figure 2:**
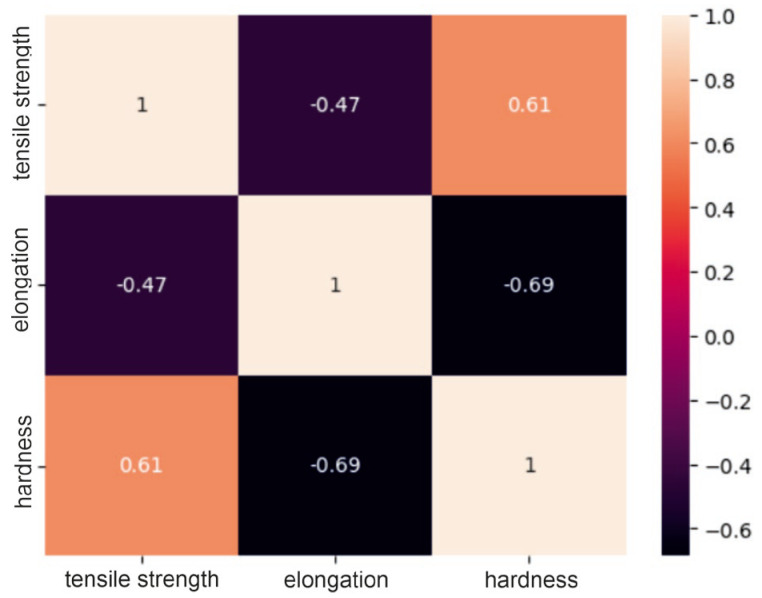
Correlation matrix for the nickel/cobalt powder group.

**Figure 3:**
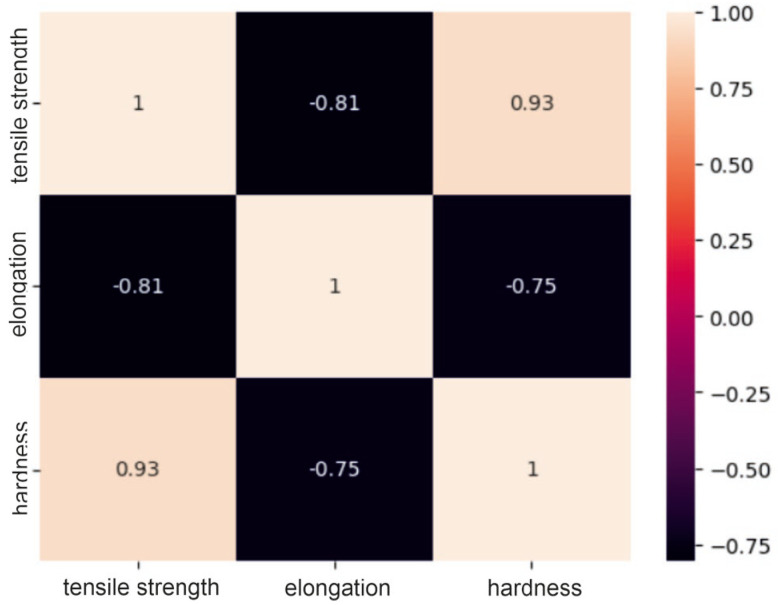
Correlation matrix for a group of steel powders.

The obtained correlation matrices for the group of steel powders based on nickel or cobalt and the group of powders based on aluminum are presented in Figure 1, Figure 2 and Figure 3.

Analysis of the correlation matrices shown above allows us to conclude that for each group of powders, there are visible relationships between the tested mechanical properties. For the group of “steel” powders, a very strong positive correlation can be observed between tensile strength and Vickers hardness. It is as much as 0.93, which means that usually high-strength materials are characterized by high hardness. There is quite a strong negative relationship between strength and elongation, amounting to −0.81. This may indicate that higher tensile strength may lead to lower elongation of the material and vice versa. A similar relationship exists between elongation and hardness. In the group of powders based on nickel or cobalt, there is a moderate positive relationship between tensile strength and hardness (0.61), a moderate negative relationship between hardness and elongation (0.69) and a moderate negative relationship (−0.47) between strength and elongation. These correlation values between mechanical properties indicate smaller dependencies between the analyzed features compared to the group of steel powders, but they are still important and are worth considering in analyses and decisions regarding materials. For aluminum-based powders, there is a fairly strong positive correlation (0.75) between tensile strength and hardness and, at the same time, a fairly strong negative correlation (−0.57) between elongation and hardness. For these powders, it can be seen that there is a weak negative relationship between their strength and elongation, which is −0.38. These results indicate that for these materials, higher strength is often associated with higher hardness, as in the previous two groups, but the relationship between strength and elongation is less clear.

### 2.2. Data Visualization: Box Plot and Histogram

**Figure 4:**
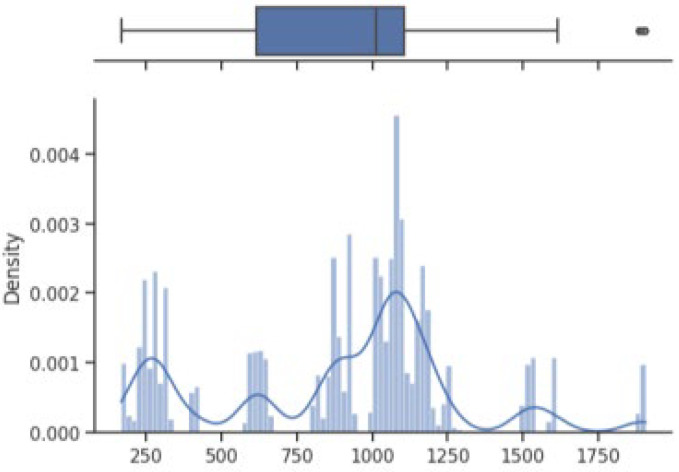
Tensile strength—boxplot and histogram.

**Figure 5:**
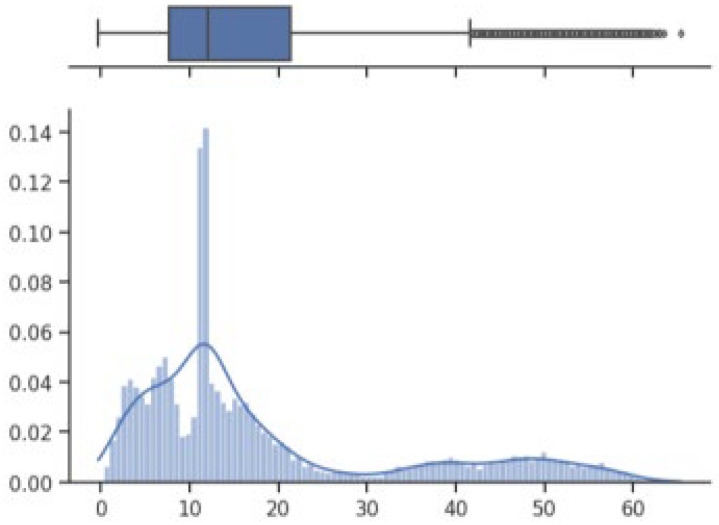
Extension—boxplot and histogram.

**Figure 6:**
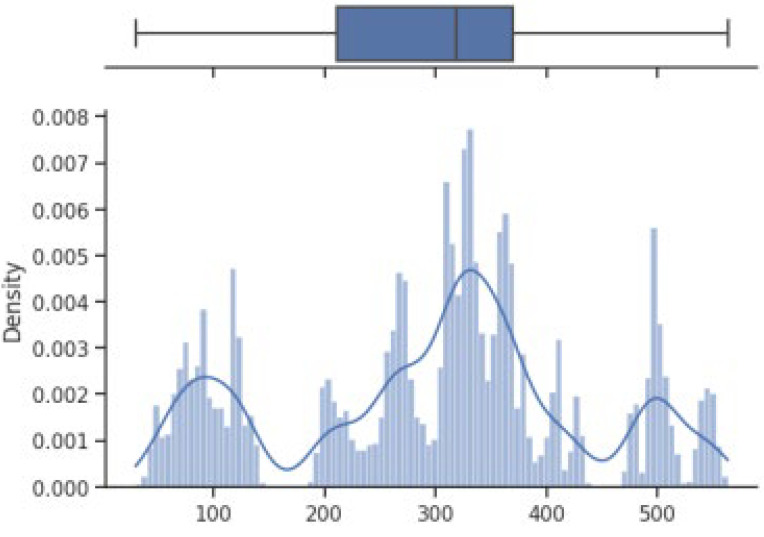
Vickers hardness—boxplot and histogram.

**Figure 7:**
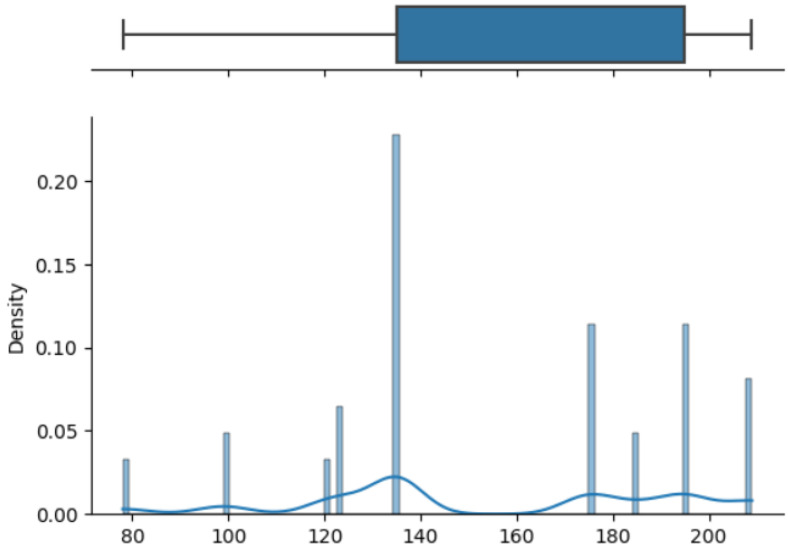
Price—boxplot and histogram.

The box on the chart indicates the area between the first quartile Q1 and the third quartile Q3, with a length equal to the InterQuartile Range—IQR. This illustrates the concentration of data, and its length corresponds to 50% of all data. The vertical line inside the box is the median, which allows you to determine the skewness of the distribution. Additionally, outside the box, there are the ends of the lines on both sides (at distances equal to Q1—1.5 IQR and Q3 + 1.5 IQR), which constitute the boundaries for non-outlier values. Outliers are marked with dots. Below each boxplot is a histogram: a graph showing the frequency of data in 100 equidistant bins. The *Y* axis for the histogram is density, and it is scaled so that the area under the bar graph sums to 1. Additionally, the approximate probability density function is marked with a solid line. Both charts share the *X*-axis, which shows the variable values. The data presented is combined synthetic data. They were sampled from 47 different normal distributions; hence, the histograms contain numerous local maxima and minima. Unlike usual in statistical analysis, outliers should also be interpreted, not as errors in the data, but rather as parameters of those powders that can give relatively different mechanical values than others. Result is shown Figure 4, Figure 5, Figure 6 and Figure 7.

How it looks on Figure 4, in case of strength, there are many minima with gaps that are not covered by any of the powders (for example, they are visible for the values of 500 and 750 MPa). These gaps mean that we will not find the correct powder recommendation for these values because there are no such products in the collected data. The central value is clearly shifted to the right, which results from the high density of data in the range from 1000 to 1250. Analyzing the figure showing the elongation characteristics, it can be concluded that the distribution of this variable is more continuous in relation to the tensile strength. Values range from 0 to 60%. The boxplot shows left-sided skewness with a median value of around 10%. Many outliers can be interpreted as parameters of powders operating in less standard ranges. In the case of Vickers hardness, two breaks can be observed for values around 175 and 450 HV. Otherwise, the distribution is continuous throughout the range with extremes from approximately 30 to 550 HV. The distribution is slightly skewed to the right; the median value is approximately 320 HV.

## 3. Results

### 3.1. Input Data Generation

The input data describing the mechanical properties—tensile strength, elongation and Vickers hardness—are presented in the form of ranges (ranges) (Table 1). For this reason, it was necessary to transform the given intervals into appropriate values, i.e., generate random data from the intervals. This process is essential when using selected machine learning algorithms and providing them with appropriate training and testing data. Two approaches were considered when generating input data:

Randomizing values from a uniform distribution and then applying data augmentation.

A uniform distribution (also known as a rectangular or flat distribution) states that each value in the interval has the same probability of occurrence; the distribution function is constant.

### 3.2. Choice of Solution

**Figure 8:**
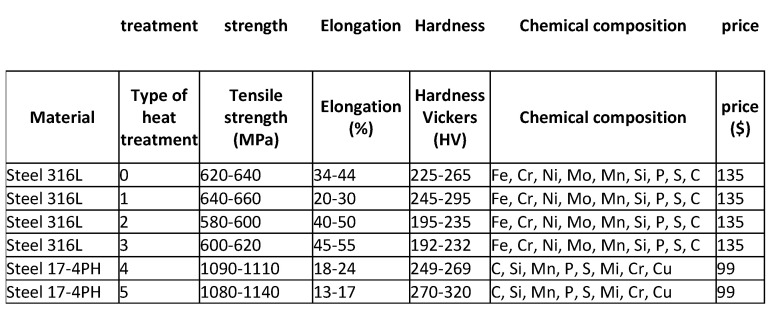
Loaded input data.

After analyzing the input data, it was decided to use an approach that draws data from a normal distribution. The reason for this choice was that the nature of the input data, i.e., the mechanical properties, were given in the form of ranges, and these ranges were determined based on experiments conducted in which the average values of the results obtained, as well as the standard deviation, were calculated. The information regarding the mean and standard deviation indicates that a better solution for generating data is to use the normal distribution. The paper assumes a distribution for which the parameter range is equal to 2 sigms. Thus, 95% of the data within the range was sampled, with 5% of the values out of the range allowed. The randomValue() method accepts parameters: 31—“lo”—lower limit of the range from which data is drawn—“hi”—upper limit of the range from which data is drawn—“num”—number of values to be drawn. Fragment of the input data is shown in Figure 8.

### 3.3. Division of Data into Training and Testing Sets

#### Description of Experiments

The following machine learning models were used in this work:

- Random Forest, an algorithm implemented from the sklearn library [24],
- Decision Tree algorithm implemented from the sklearn library [25],
- GradientBoost algorithm implemented from the sklearn library [26],
- XGBoost algorithm implemented from the xgboost library [27],
- AdaBoost algorithm implemented from the sklearn library [28],
- K-Nearest Neighbors, an algorithm implemented from the sklearn library [29],
- Fuzzy K-Nearest Neighbors, own implementation.

Attempts were made to use various machine learning models to check which of them works best in classifying this type of data. The decision to use specific machine learning algorithms was related to the characteristics of the problem and the benefits that these models can provide. Models with boosting and gradient boosting techniques (XGBoost, GradientBoost, AdaBoost) were chosen primarily because they often achieve very high prediction accuracy.

### 3.4. Hyperparameter Tuning—Testing the Best Configurations

In the case of algorithms from the sklearn library, it is possible to improve their performance by tuning hyperparameters. This library enables the use of tools such as GridSearchCV or RandomsizedSearchCV, which automatically searches the hyperparameter space to find the best combinations for a given model. Hyperparameters are variables that control the model learning process and, when properly selected, influence the operation of algorithms, improving their prediction quality. The sets from which the best hyperparameters were searched also included default model parameters. In the case of the Fuzzy K-Nearest Neighbors algorithm, the default parameters reflected those used when writing our own implementation.

- Selected hyperparameters and values for the Random Forest algorithm
  ○ n_estimators [50, 100, 200]—number of trees (estimators) in the algorithm,
  ○ max_depth [None, 10, 20]—maximum tree depth (overfitting control),
  ○ min_samples_split [2, 5, 10]—the minimum number of samples required to split a node in the tree.

- Selected hyperparameters and values for the Decision Tree algorithm
  ○ max_depth [None, 10, 20, 30]—maximum tree depth (overfitting control),
  ○ min_samples_split [2, 5, 10]—minimum number of samples required to split a node in the tree,
  ○ min_samples_leaf [1, 2, 5]—the minimum number of samples required to create a leaf in the tree.

- Selected hyperparameters and values for the XGBoost algorithm
  ○ n_estimators [50, 100, 200]—number of trees (estimators) in the algorithm,
  ○ max_depth [3, 5, 7]—maximum tree depth (overfitting control),
  ○ learning_rate [0.01, 0.1, 0.2]—learning rate, determines how large the weight update step is at each stage.

- Selected hyperparameters and values for the AdaBoost algorithm
  ○ n_estimators [50, 200, 500, 700, 900, 1000]—number of trees (estimators) in the algorithm,
  ○ learning_rate [1.0, 0.2, 0.3, 0.4, 0.5, 0.7]—learning rate, determines how large the weight update step is at each stage. For the AdaBoost model, the number of parameters is larger than in the case of other models, because in this case the search range has been extended.

- Selected hyperparameters and values for the GradientBoost algorithm
  ○ n_estimators [50, 100, 200]—number of trees (estimators) in the algorithm,
  ○ max_depth [3, 5, 7]—maximum tree depth (overfitting control),
  ○ learning_rate [0.01, 0.1, 0.2]—learning rate, determines how large the weight update step is at each stage.

- Selected hyperparameters and values for the K-Nearest Neighbors algorithm
  ○ n_neighbors [3, 5, 7]—number of neighbors in the algorithm
  ○ weights [‘uniform’, ‘distance’]—method of assigning weights, where: “uniform”—equal weights for all neighbors, “distance”—weights inversely proportional to the distance
  ○ *p* [1, 2, 3]—parameter defining the norm for calculating the distance between neighbors, where: *p* = 1—Manhattan norm, *p* = 2—Euclidean norm, *p* = 3—Minkowski norm.

- Selected hyperparameters and values for the Fuzzy K-Nearest Neighbors algorithm o k [3, 5, 6]—the number of nearest neighbors taken into account when classifying a new point, 42 o m [1.5, 2.0, 3.0, 4.0]—the degree of belonging of the point to different classes. The value of m″ determines how fuzzy the affiliations should be—as the value of m″ increases, the affiliations become more evenly distributed, and the influence of further points is weaker. In the project, hyperparameter tuning was performed using Grid-SearchCV using 5-fold cross-validation. GridSearchCV is a better choice when you have a small hyperparameter space to tune. Unlike RandomsizedSearchCV, it searches all possible combinations and allows you to provide the most accurate results. Thanks to the use of cross-validation in GridSearchCV, the results of model evaluation for various combinations of hyperparameters are more reliable because the algorithm is tested on different data divisions (folds), which eliminates the phenomenon of randomness of training and testing data and ensures better ability for generalization. The f1-score evaluation measure was used to assess the quality of models in the Grid Search process.

### 3.5. Extension of Hyperparameter Ranges

When testing the best configurations for the AdaBoost model, the following ranges were initially searched for hyperparameters: o learning_rate [0.1, 0.2, 0.3] on_estimators [50, 200, 500]. The algorithm achieved the best results for the extreme values of the tested parameters: learning_rate = 0.3 and n_estimators = 500. Therefore, it was decided to continue the study to find parameters that would give even better results. For this reason, new tested hyperparameter values were selected for the AdaBoost algorithm:

- learning_rate [0.3, 0.5, 0.7]
- n_estimators [500, 700, 900, 1000]. After conducting the study using the GridSearchCV technique, the best values of the tested hyperparameters in terms of the f1-score metric turned out to be:
- learning_rate = 0.5
- n_estimators = 900 43

Table 2 compares the metrics results for the AdaBoost algorithm obtained in two hyperparameter optimization studies on the test set. The best algorithm settings for both tests are:

− First study: learning_rate = 0.3, n_estimators = 500
− Second study: learning_rate = 0.5, n_estimators = 900

Based on the results in Table 2, it can be concluded that continuing the research to find better hyperparameters turned out to be right because the results obtained for all metrics improved. Because the new best hyperparameters were not the extreme parameters of the searched intervals, no further examination continued, and these results were considered as the best possible for this model.

### 3.6. List of the Best Values for the Tested Hyperparameters

Testing the configuration of hyperparameters made it possible to find those that, when combined, provide the best results, that is, the highest value of the f1-score metric. Table 3 lists the most optimal hyperparameter configurations for each model. They were determined using the GridSearchCV technique on the training set.

### 3.7. Evaluating the Quality of Prediction Models for the Best Parameters

Using the best hyperparameter configurations, the performance of the predictive models was assessed. To avoid overfitting the hyperparameters to the training data, the models were evaluated on an independent test set. The use of multithreading was possible thanks to the n_jobs parameter, which is available in selected implementations of machine learning algorithms.

### 3.8. Experimental Results

#### Cross-Validation of Models—Results and Conclusions

In the beginning, it should be noted that the results achieved concern synthetic data, which is generated from distributions based on parameters taken from the publication. The assumption that all powder characteristics are distributed according to a normal distribution cannot be met in reality, but in this work, it was necessary to achieve similar powder recommendation results and the obtained results should be treated as such. Additionally, for real data, we often deal with unbalanced classes, which significantly worsens the results. This situation does not occur in this work either, because the number of generated examples was the same for all classes. Table 4 contains the cross-validation results.

### 3.9. Conclusions for the Results Obtained after Cross-Validation of the Models

It can be concluded that the results obtained are high for each of the models except the AdaBoost model, which did not match without tuning, which may be indicated by the F1 metric = 8.13%. The results for the remaining algorithms reach values above 98% for all metrics, which proves very high classification efficiency. The best results for each of the tested metrics were obtained for the Random Forest model, 98.66%, recall (sensitivity), with a deviation of 0.0025. The result of a single tree (Decision Tree algorithm) compared to Random Forest is not surprising and, as expected, is lower (98.13%). The K-Nearest Neighbors, Fuzzy K-Nearest Neighbors, XGBoost and Gradient Boost models performed slightly lower, but the differences are only the third decimal place, indicating that the errors are within the statistical error range. In the case of the KNN model, which is based on the calculation of the Euclidean distance, for classes that are more difficult to separate, errors may result from small differences in the distances from the generated points. The difference between the version with the Fuzzy KNN algorithm and the standard version is negligible. The motivation for using fuzzy logic (using the Fuzzy KNN algorithm) was the expectation of improving performance, especially for overlapping classes, which was not achieved. To sum up, based on the results, it can be concluded that all selected algorithms, except the AdaBoost algorithm, achieve high results, which indicates that they are effective in classification; additionally, low values of standard deviation indicate the stability of the results. A slight advantage of one model over another may mean that for the default parameters this model achieves the best results, but after adjusting the hyperparameters, one of the other tested models could prove to be better. In the case of hyperparameter tuning, the set was previously divided into test and training. Cross-validation was performed on the training set, which was used in the GridSearch algorithm to validate which set of parameters was the best. However, at the very end, it was tested on the test set. This eliminates the problem of overfitting models to hyperparameters. However, it should be noted that the results obtained before and after hyperparameter tuning performed on the test set cannot be compared with the results from cross-validation. The obtained results only provide an overview for formulating general conclusions about the differences between the tested models. For the results obtained before and after hyperparameter tuning, when comparing the results, the main focus was on the recall and f1-score metrics, because they provide a comprehensive look at the model performance and facilitate the interpretation of the results. Outside the AdaBoost model, differences before and after tuning only occur at the level of three or even four significant digits, which may suggest that the change is statistically insignificant. To better visualize the results, a good solution would be to present how the results change before and after tuning per class. Unfortunately, due to the large number of classes (47) and tested models (7), the results are not attached, but it should be noted that such a study was carried out and the results indicated that the models before tuning achieved the F1 value for the majority of classes equal to 1. This means that the results after tuning only for less than half of the tested classes could be improved. Therefore, the improvement or deterioration of results for single labels may be underestimated. To highlight changes in individual classes, those classes for which tuning did not reduce the value of F1 = 1.0 were not considered. Analyzing the results, the greatest improvement can be seen for the AdaBoost model, by over 67% for the f1 metric. On this basis, it can be concluded that for the examined problem, this algorithm is very sensitive to hyperparameters. However, it can still be concluded that this model performs much worse than the other tested algorithms, and its results are still far from satisfactory for this type of problem. Perhaps additional parameters should be added: base_estimator (base model—accepting the values: decision tree (default), SVM and logistic regression) or algorithm (algorithm for calculating sample weights), which could improve its performance. For the remaining tested models, no significant improvement was achieved (the difference did not exceed 1 percentage point). In the case of Random Forest and Decision Tree—the improvement on the F1 metric was about 0.70%, and for the XGBoost algorithm—0.45%. For the Gradient Boost, KNN and Fuzzy KNN models, the change does not exceed two-thousandths of a percent. Such low results prove that the generated data set can be classified with very high efficiency even without tuning. However, there are samples in the data that lie close to the multiclass decision boundary (powders with overlapping parameters). In this case, parameter optimization will not produce the desired result, and a specific set of parameters may correspond to multiple correct powder recommendations. The assumption that all powder characteristics are distributed according to a normal distribution cannot be met in reality, but in this work, it was necessary to achieve similar powder recommendation results and the obtained results should be treated as such. Additionally, we often deal with unbalanced classes, which significantly worsens the results.

### 3.10. Decision-Making System Application

The research concept is based on the creation of a decision support tool that indicates appropriate material based on data entered by the user. Therefore, it was necessary to create an application with a graphical user interface. To create a decision-making system, the Python library, Streamlit was used, thanks to which it is possible to create interactive web applications. The application has an interface only in Polish. A key element that significantly contributed to increasing the application’s performance was the use of .pkl files in which the results of previously trained machine learning models were saved.

**Figure 9:**
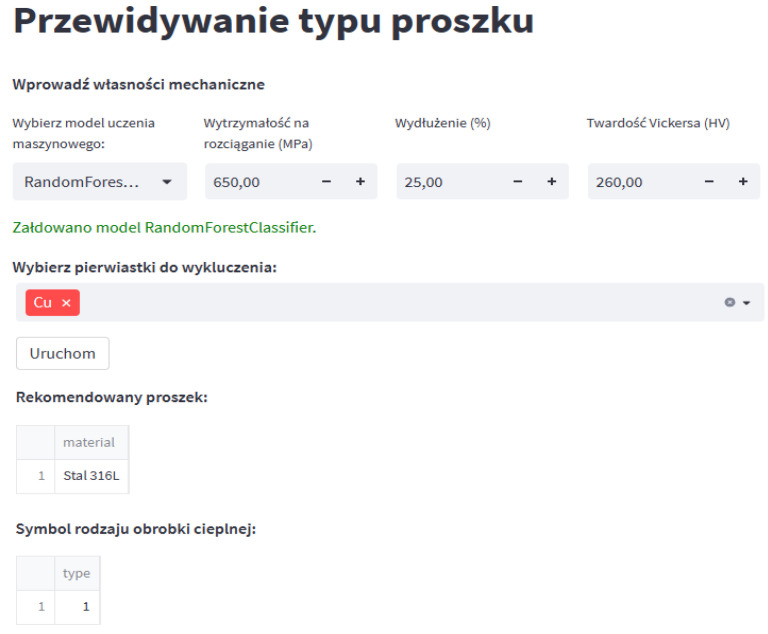
Graphical user interface of the created decision-making system.

The use of PKL files is a common practice when using Python because it enables serialization, i.e., transforming a data structure into a form that can be easily saved and then recreated. Saving the results of trained models in this form allows you to avoid the need to repeat the model training process each time you run the application. The Python language has a built-in ‘pickle’ module, which provides methods for writing data in the appropriate form—pickle.dump() and reading data—pickle.load(). The created graphical interface is presented in Figure 9. Thanks to it, the user can enter appropriate mechanical properties, such as tensile strength, elongation and Vickers hardness, and can also select a machine learning model, based on which the system will predict the best powder. Additionally, the user can select undesirable chemical elements from the list, which means that powders that contain them in their chemical composition will not be considered. Using the “Run” button, the user starts the process of searching for the recommended powder and heat treatment, and the application returns the result.

**Figure 10:**
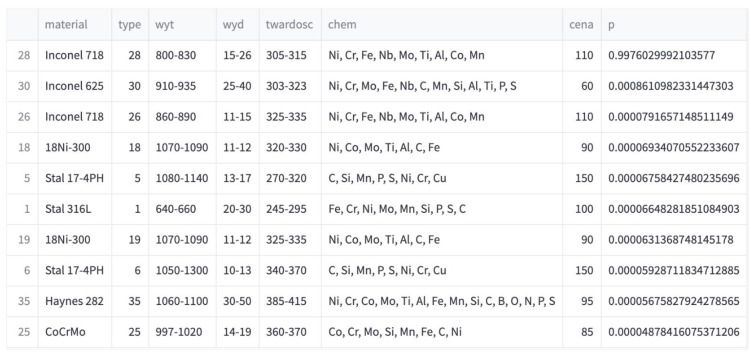
Example result of the functionality of creating a ranking of the 10 best powders.

Additionally, in addition to predicting the most suitable powder, the application also builds a ranking of the 10 best matches. For this purpose, the predict_proba() method from the sklearn library was used, which returns an array with the probabilities of belonging to each class. The data in the table is additionally enriched with information on the original parameter ranges, price and chemical composition of the powders, and the results are sorted in descending order. Figure 10 shows an example ranking result of the top 10 matches.

Such a ranking is a useful functionality in which at the end you can assess for how many items in the ranking all parameters fall within the desired ranges. The last column contains the probability of belonging to a specific class. It is worth adding that after filtering the elements, the probability values may be very low because the most probable items have been removed due to their chemical composition. In such a case, the given ranges for each parameter should be used to assess whether any of them have been exceeded or whether the exceedance is acceptable.

## 4. Discussion and Conclusions

**Figure 11:**
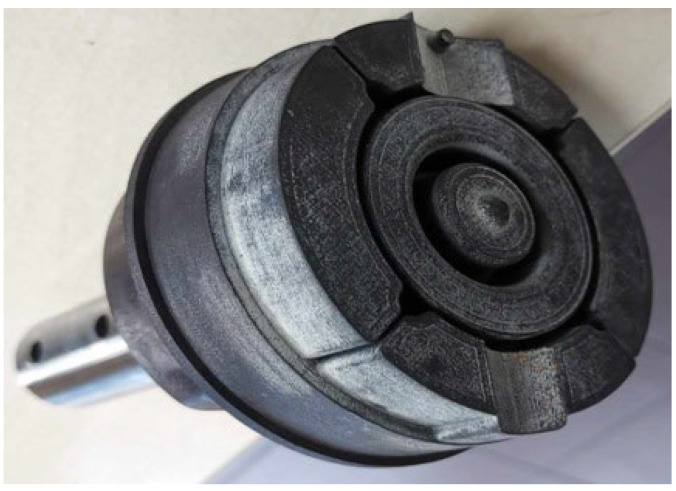
An example of a core made using 3D printing is shown in figure.

The aim of the work, which was to investigate the capabilities of machine learning classifiers in the issue of recommending powders for 3D printing, was achieved. The work uses cross-validation as well as hyperparameter tuning using different sets for evaluation; the entire set was used in the first case and a separate test set was used in the second. Therefore, different results were achieved, but in both cases, the best model turned out to be Random Forest, whose F1 metric score is 98.66% for cross-validation and 99.10% after tuning on the test set. Therefore, this model can be considered the most promising 51 for the problem posed in this way. However, both results cannot be compared; the first one is a more accurate estimate of how the model will behave for new data, while the second one talks about possible improvement after optimization or possible overfitting to the parameters. In a production application, it is recommended to use the model that turned out to be the best (Random Forest with the hyperparameters found: n_estimators = 50, max_depth = None, min_samples_split = 2). Then, the estimated accuracy of the model should not be lower than the cross-validation result obtained during testing, which in practice means that the application will operate on new data as expected and with high accuracy. Information regarding the validation results and search parameters may be included in the user’s documentation, but changing these parameters is not within the competence of the technologist using the application, because he or she does not know this area. The applied approach of generating synthetic data is a good solution for building a base version of the decision-making system, using knowledge from independent research. This is a beneficial option when actual data is limited or difficult to access. However, it should be remembered that for real data, the results would certainly deteriorate, and the assumption regarding the normal distribution of parameters may seem to be a significant simplification, but it constitutes the basis and gives the possibility of future enrichment of the generated data with more precise information about the distributions or using actual measurement data for classification. Nevertheless, even now, by using the created application that builds a ranking of the best powders concerning the expected use of the product, we can build an effective recommendation system using validation based on input ranges and, as a result, selecting the product with the most appropriate price. Verification of the operation of this solution was carried out in relation to the selection of material for operation in conditions of a variable temperature field, e.g., in molds used in pressure casting. For such an application, the choice of steel is, e.g., H10, H13 [30]. In this case, the material should have medium mechanical properties [31]: hardness 46 HRC, Rm = 1200 MPa, A = 8%. Based on the defined parameters, the system proposed the selection of a material, in this case, 18Ni300 powder, which will be used to produce a core reproducing the shape of the casting using additive technology. Such an example element was made and is presented in Figure 11.

Material tests carried out on the printed material allowed for the determination of the final mechanical properties at the level of Rm = 1650 MPa, A = 8.4%, HRC 48.5.

## Figures and Tables

**Table 1:** Example of data.

Material	Type of Processing	Tensile Strength [MPa]	Elongation [%]	Vickers Hardness [HV]
Steel 316L	no	620–640	34–44	225–265
	600 °C, 2 h, air cooling	640–660	20–30	245–295
	950 °C, 2 h, air cooling	580–600	40–50	195–235
	1095 °C, 2 h, water cooling	600–620	45–55	192–232
Steel 17-4PH	Brak	1090–1110	18–24	249–269
	1040 °C, 0.5 h, air cooling	1080–1140	13–17	270–320
	550 °C, 4 h, air cooling	1050–1300	10–13	340–370
18Ni-300	No	1240–1260	11–12	375–385
	750 °C, 6 h, Argon atmosphere	1180–1200	11–12	355–365

**Table 2:** Comparison of metric results for hyperparameter optimization.

Metryka	First	Second	Difference
Accuracy Test	0.6643	0.7094	+0.0451
Precision Test	0.6321	0.7068	+0.0747
Recall Test	0.6643	0.7093	+0.0450
F1-score Test	0.6157	0.6530	+0.0373
MCC Test	0.6686	0.7074	+0.0388

**Table 3:** The best hyperparameter values for the tested machine learning models.

Algorithm	The Best Values of Selected Hyperparameters			f1-Score
Random Forest	n_estimators	max_depth	min_samples_split	0.9816
	50	None	2	
Decision Tree	max_depth	min_samples_split	min_samples_leaf	0.9734
	20	10	1	
XGBoost	n_estimators	max_depth	learning_rate	0.9692
	50	3	0.2	
AdaBoost	learning_rate		n_estimators	0.6530
	0.5		900	
Gradient Boost	learning_rate	max_depth	n_estimators	0.9795
	0.1	3	50	
K-Nearest Neighbors	n_neighbors	weights	*p*	0.9774
	7	distance’	1	
Fuzzy KNN	k		m	0.9831
	3		3.0	

**Table 4:** Cross-validation results for the tested machine learning models.

Model	Mean Accuracy	Std. Accuracy	Mean Recall	Std. Recall	Mean Precision	Std. Precision	Mean F1	Std. F1
Random Forest	0.9867	0.0025	0.9867	0.0025	0.9870	0.0024	0.9866	0.0025
Decision Tree	0.9813	0.0034	0.9813	0.0034	0.9818	0.0035	0.9813	0.0034
XGBoost	0.9812	0.0034	0.9812	0.0034	0.9816	0.0033	0.9813	0.0034
AdaBoost	0.1237	0.0362	0.1237	0.0362	0.0782	0.0279	0.0830	0.0285
Gradient Boost	0.9810	0.0040	0.9810	0.0040	0.9818	0.0040	0.9811	0.0040
KNN	0.9832	0.0024	0.9832	0.0024	0.9841	0.0025	0.9830	0.0025
Fuzzy KNN	0.9842	0.0021	0.9842	0.0021	0.9835	0.0020	0.9830	0.0022

## Data Availability

The data presented in this study are available upon request from the corresponding author.
